# Electrode Humidification Design for Artifact Reduction in Capacitive ECG Measurements

**DOI:** 10.3390/s20123449

**Published:** 2020-06-18

**Authors:** Yue Tang, Ronghui Chang, Limin Zhang, Feng Yan, Haowen Ma, Xiaofeng Bu

**Affiliations:** School of Electronic Science and Engineering, Nanjing University, Nanjing 210046, China; dg1723024@smail.nju.edu.cn (Y.T.); mf1823002@smail.nju.edu.cn (R.C.); fyan@nju.edu.cn (F.Y.); hwma@nju.edu.cn (H.M.); xfbu@nju.edu.cn (X.B.)

**Keywords:** humidification design, capacitive ECG, textile electrode, triboelectric charge, motion artifact

## Abstract

For wearable capacitive electrocardiogram (ECG) acquisition, capacitive electrodes may cause severe motion artifacts due to the relatively large friction between the electrodes and the dielectrics. In some studies, water can effectively suppress motion artifacts, but these studies lack a complete analysis of how water can suppress motion artifacts. In this paper, the effect of water on charge decay of textile electrode is studied systematically, and an electrode controllable humidification design using ultrasonic atomization is proposed to suppress motion artifacts. Compared with the existing electrode humidification designs, the proposed electrode humidification design can be controlled by a program to suppress motion artifacts at different ambient humidity, and can be highly integrated for wearable application. Firstly, the charge decay mode of the textile electrode is given and it is found that the process of free water evaporation at an appropriate free water content can be the dominant way of triboelectric charge dissipation. Secondly, theoretical analysis and experiment verification both illustrate that water contained in electrodes can accelerate the decay of triboelectric charge through the free water evaporation path. Finally, a capacitive electrode controllable humidification design is proposed by applying integrated ultrasonic atomization to generate atomized drops and spray them onto textile electrodes to accelerate the decay of triboelectric charge and suppress motion artifacts. The performance of the proposed design is verified by the experiment results, which shows that the proposed design can effectively suppress motion artifacts and maintain the stability of signal quality at both low and high ambient humidity. The signal-to-noise ratio of the proposed design is 33.32 dB higher than that of the non-humidified design at 25% relative humidity and is 22.67 dB higher than that of non-humidified electrodes at 65% relative humidity.

## 1. Introduction

It is well known that, with the increasing demand for personal daily health monitoring, the study of wearable bioelectrical acquisition equipment is becoming increasingly popular. The non-contact bioelectrical acquisition equipment is one kind of wearable bioelectrical acquisition equipment. Compared with the traditional contact acquisition equipment using the wet electrodes (i.e., Ag/AgCl electrodes), it will not cause skin irritation and allergic contact dermatitis [1], and does not need detailed skin preparation [2], which can meet the requirement of long-term bioelectricity acquisition [3,4]. The non-contact electrodes belong to capacitive electrodes, which acquire the signal through clothes or some dielectrics [5]. The motion artifacts are caused by the triboelectricity generated by friction and the variation of the skin–electrode interface [5,6]. Therefore, non-contact electrodes may cause severe motion artifacts due to the relatively large friction between the electrodes and the dielectrics, which is also an important reason why the non-contact bioelectrical acquisition equipment is difficult to use in clinical applications [5,7]. 

In the current research, there are three kinds of methods to suppress motion artifacts. The first method is to design special electrodes. Weder et al. [8] and Leicht et al. [9] proposed the designs of humidifying electrode with water tank. Fong and Chung proposed a design of a hygroscopic electrode with superabsorbent polymer [10]. Wartzek et al. [7] and Peng et al. [11] proposed the designs of electrode with grids to discharge the triboelectric charge. Park′s group proposed the flexible electrodes with conductive polymer foam to reduce the relative movement of the electrodes to the skin [12,13,14]. The second method is to use reference signal with correlation for artifact reduction. Serteyn et al. used an injection signal to track the changes of coupling capacitance and estimate motion artifacts [15]. Choi et al. used the acquisition signal of an adjacent non-intrusive electrocardiogram (ECG) sensor as reference signal to suppress motion artifacts [16]. Yoon et al. [17] and Ottenbacher et al. [18] estimated the motion artifacts based on the motion data of acceleration motion sensors. The third method is based on an algorithm to suppress motion artifacts. Lee et al. used the wavelet approach to remove motion artifacts from the ECG signal [19]. Salehizadeh et al. reconstructed the heart rate signal corrupted by motion artifacts based on a novel time-varying spectral filtering algorithm [20]. Dey et al. [21] and Poungponsri et al. [22] used the artificial neural network approach to suppress various noises in ECG signals. All of the above three methods can suppress motion artifacts to a certain extent, where the first method is based on the generation mechanism of motion artifacts and does not need a complex algorithm or processing circuit. A better signal-to-noise ratio can be obtained with artifact reduction based on the generation mechanism of motion artifacts [6,10]. However, there are still some limitations in the existing research of the first kind of method. In the existing artificial humidification design, resistance heating wires were used to make moisture evaporation, which required a large volume and high power and were not suitable for wearable applications [9]. In the existing natural humidification design, the process of electrode humidification is the natural infiltration process of water vapor in a water tank or environment, and this process is not controllable and has low effectiveness [8,10]. Moreover, the existing humidification designs all lack a complete analysis of how water can suppress motion artifacts and the effect of water is only considered to reduce the impedance of the skin–electrode interface. The designs of elastic electrodes have ignored the influence of the triboelectricity in a dry environment [12,13,14].

For wearable capacitive ECG acquisition, capacitive electrodes may cause severe motion artifacts due to the relatively large friction between the electrodes and the dielectrics. In this paper, the effect of water on charge decay of textile electrode is studied systematically, and a capacitive electrode controllable humidification design is proposed to suppress motion artifacts. Compared with the existing electrode humidification designs, the proposed design can be controlled by a program to humidify the electrode, which can effectively suppress motion artifacts at different ambient humidity, and can be highly integrated for wearable applications. Firstly, the charge decay model of the textile electrode is given to illustrate the dominant way of triboelectric charge dissipation. Secondly, both theoretical analysis and experiment results have shown that water contained in electrodes can accelerate the decay of triboelectric charge through the free water evaporation path (FWEP). Thirdly, a capacitive electrode humidification design is proposed by applying integrated ultrasonic atomization to generate atomized drops and spray them onto textile electrodes to accelerate the decay of triboelectric charge and suppress motion artifacts. Finally, the performance of the proposed design is verified by the experiment results.

## 2. Charge Decay Model of Textile Electrode

A textile electrode, as flexible electrodes, is often used in wearable bioelectricity monitoring [23,24,25]. When it is used as a capacitive electrode, the friction among skin, fabric and electrode due to motion causes the charge accumulation on the textile electrode [26]. Generally, the surface charge of charged materials will decay with time. In some research, there are two main ways of charge decay: Charge leakage and charge neutralization [27,28]. However, most of the materials tested in these studies were hydrophobic insulators and those materials were connected to the earth directly. For hydrophobic insulator materials, the way of charge leakage includes electric conduction through the volume of the insulator and electric conduction through the surface of the insulator [27]. For hydrophilic textile materials, the way of charge leakage also includes the process of charge escape (charge escapes into the air through the free water evaporation path) [29,30]. 

**Figure 1:**
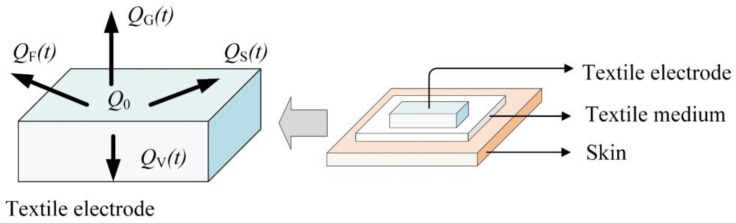
Schematic diagram of charge decay model of a charged capacitive textile electrode.

Figure 1 is a schematic diagram of a charge decay model of a charged capacitive (non-contact) textile electrode, where *Q*_0_ is the initial charge amount of the electrode, *Q*_V_(*t*) is the charge decay through volume conduction path, *Q*_S_(*t*) is the charge decay through surface conduction path, *Q*_G_(*t*) is the charge decay through gas ions neutralization path and *Q*_F_(*t*) is the charge decay through FWEP.

The residual charge on the textile electrode *Q*(*t*) can be expressed as in Equation (1).

(1) $$Q(t)=Q_{0}-\left[Q_{V}(t)+Q_{S}(t)+Q_{G}(t)+Q_{F}(t)\right]$$

It should be noted that the analysis of charge decay through the surface conduction path is relatively complex. Simulation studies have shown that during the decay charge through the surface conduction path, the total charge on the surface of the material remains constant until the charge reaches the edge of the material and can flow to the earth through some other paths [27], and the charge is usually transferred from insulating material to conductive material with low resistance [26]. Therefore, for the charge decay on the conductive textile electrode, only three paths, the volume conduction path, the gas ions neutralization path and the FWEP, are considered in this paper.

**Figure 2:**
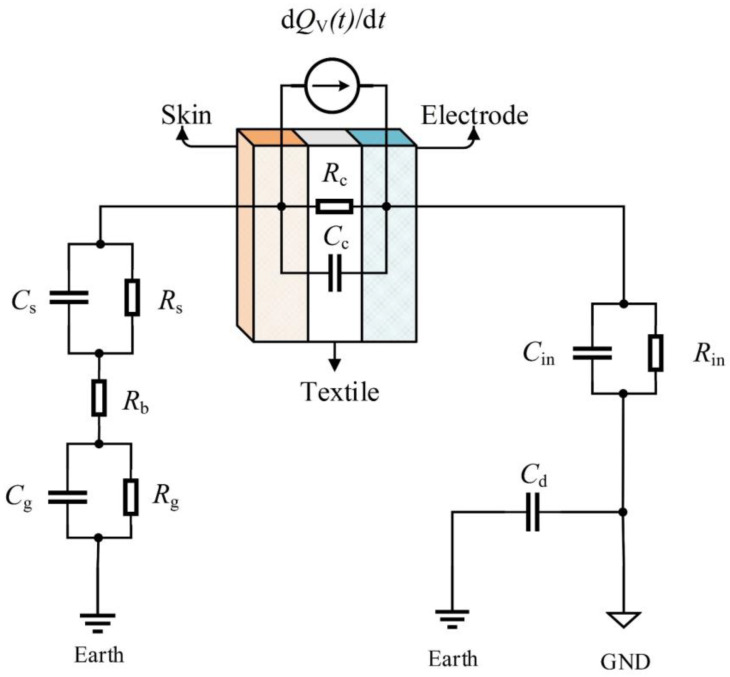
The charge decay circuit model of charge leakage to the earth from a textile electrode.

When only considering the volume conduction path, the charge decay circuit model of charge leakage to the earth from textile electrode is shown in Figure 2, where *R*_c_ and *C*_c_ are the equivalent resistance and capacitance of skin–electrode interface, *R*_s_ and *C*_s_ are the resistance and capacitance of stratum corneum, *R*_g_ and *C*_g_ are the resistance and capacitance of body against earth, *R*_b_ is the body equivalent resistance, *R*_in_ and *C*_in_ are the equivalent input impedance and capacitance of the acquisition front end and *C*_d_ is the distribution capacitance between the signal ground and the earth of the floating system. To simplify the model, the possible capacitive driven-right-leg (C-DRL) circuit and C-DRL electrode for common mode interference suppression have not been considered in Figure 2. In fact, the severe artifacts will still occur, although a good connection of C-DRL exists [7].

The residual charge on the textile electrode *Q*′_V_(*t*) can be expressed by Equation (2), where *τ*_v_ is the time constant of the volume conduction path. The complete expression of *τ*_v_ is related to all the resistances and capacitances in the charge decay circuit model and it can be approximated to Equation (3) by using the first-order approximation method based on the numerical simulation software MATLAB.

(2) $${Q_{V}}^{\prime }(t)=Q_{0}-Q_{V}(t)=Q_{0}\cdot exp\left(-\frac{t}{\tau_{v}}\right)$$

(3) $$\tau_{v}\approx R_{\mathrm{in}}(C_{d}+C_{\mathrm{in}})+(R_{b}+R_{g}+R_{s}+R_{c})C_{d}+R_{s}C_{s}+R_{g}C_{g}+R_{c}C_{c}$$

When only considering the gas ions neutralization path, the gas ions produced by the natural background radiation in the air around the electrode will be neutralized with the charge on the textile electrode (without considering corona discharge) [27,31]. The residual charge on the textile electrode *Q*′_G_(*t*) can be expressed by Equation (4), where *Z* is the ion mobility in the air, *n* is the ion concentration in the air, *ε*_0_ is the vacuum dielectric constant, and e is an elementary charge.

(4) $${Q_{G}}^{\prime }(t)=Q_{0}-Q_{G}(t)=Q_{0}\cdot exp\left(-\frac{Znet}{\varepsilon_{0}}\right)$$

When only considering the FWEP, the residual charge on the textile electrode *Q*′_F_(*t*) can be expressed by Equation (5), where *λ* is the rate constant of the charge escaping into the air and is related to the free water content of the material (textile electrode) and the relative humidity (RH) of the air [29].

(5) $${Q_{F}}^{\prime }(t)=Q_{0}-Q_{F}(t)=Q_{0}\cdot exp(-\lambda t)$$

The parameter values of the model in Equations (2)–(5) under the environmental conditions of 20 °C and 65% RH are listed in Table 1, where the material surface composition of the textile electrode is 50% silver fiber and 50% polyester fiber [32].

**Figure 3:**
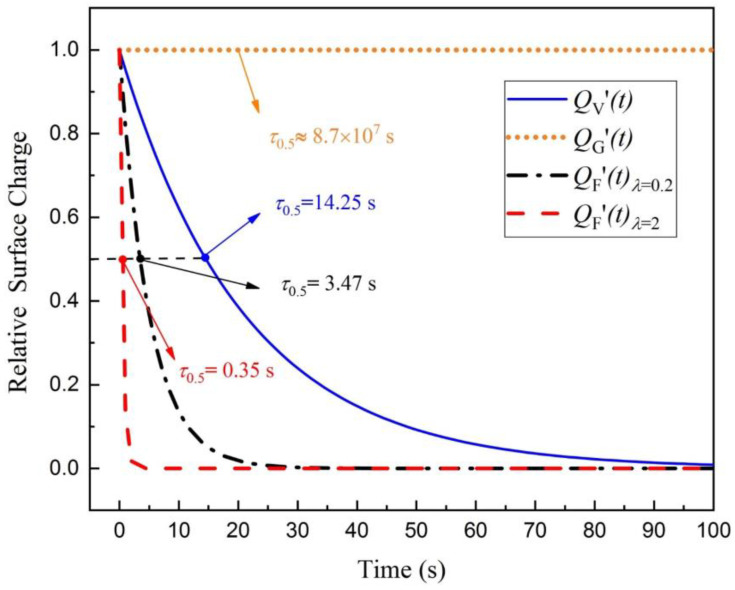
Relative surface charge with time for textile electrode through different paths. Compared with other paths, the half-life of charge decay through free water evaporation path (FWEP) can be very short.

According to the values of the parameter in Table 1, the function curves of *Q*′_V_(*t*), *Q*′_G_(*t*) and *Q*′_F_(*t*) in Equations (2)–(5) are normalized and plotted in Figure 3. Since the rate constant of charge escaping into the air is related to the free water content of the material and the relative humidity of the air, the range of *λ* measured in the experiment is about [0.07,17.43] in reference [29] and *λ* is taken as 0.2 and 2, respectively, for the simulation in Figure 3. As shown in Figure 3, the blue solid curve represents the decay of relative surface charge with time for textile electrode through the volume conduction path, the orange dot curve represents the decay of relative surface charge with time on the textile electrode through the gas ions neutralization path. The black dash–dot curve and the red dash curve represent the decay of relative surface charge with time for a textile electrode through the FWEP when *λ* is 0.2 and 2, respectively, where 0.2 is the experimental value of polyester fiber at 20 °C, 65% RH and 2 is the experimental value of cotton under high humidity [29]. The time when the charge decays to half of the initial charge is defined as half-life τ_0.5_ and labeled in Figure 3. The shorter the half-life, the shorter the stabilization time of the motion artifacts caused by charge [6].

As shown in Figure 3, assuming no corona discharge, the half-life of charge decay through the gas ion neutralization path is theoretically very long. The charge can hardly be decay through the gas ion neutralization path, while the volume conduction path and the FWEP are the main paths of charge decay. The half-life of the volume conduction path is 14.25 s in Figure 3, which may change with different parameters in some specific environment according to Equation (3). In fact, the half-life depends mainly on *R*_in_, *C*_in_ and *C*_d_, which is similar to the conclusion in reference [7]. The half-life of FWEP will change with a different rate constant of charge escaping into the air *λ*, where the half-lives of the FWEP are 3.47 s and 0.35 s when *λ* is 0.2 and 2, respectively. The larger the *λ* is, the shorter the half-life is.

In the actual situation, the effect of the above decay paths may exist at the same time, and the path with the shortest half-life will play a leading role. According to the charge decay circuit model in Figure 2, if the volume conduction path plays the leading role, most of the charge flows into the input of the acquisition front end, which may eventually cause severe motion artifacts. According to Figure 3, if the FWEP plays the leading role, most of the charge can be rapidly decayed through the FWEP rather than the volume conduction path, and the motion artifacts will be improved due to the limited charge inflow at the input of the acquisition front end. Therefore, how to make the FWEP play a leading role becomes the key to suppressing the motion artifacts caused by the triboelectricity.

## 3. The Effect of Water on the Charge Decay of Textile Electrode

### 3.1. Theoretical Analysis

Theoretically, when the environmental parameters are constant, the change of free water content in the textile electrode or the water content in the air around the textile electrode will both affect the charge decay of the textile electrode. 

For the gas ions neutralization path, the increase of the water content in the air around the textile electrode can be equivalent to the increase of local relative humidity around the electrode. The increase of the relative humidity of the air does not affect the equilibrium ion concentration *n* [38], but will lead to the decrease of the ion mobility in the air *Z* [39]. Therefore, the increase of the water content in the air around the textile electrode will not accelerate the charge decay of the gas ions neutralization path.

For the volume conduction path, the increase of free water content of the textile electrode or the water content in the air around the textile electrode may also increase the water content of the textile dielectric and reduce the equivalent impedance of the skin–electrode interface (*R*_c_||*C*_c_) [6,23], which can theoretically reduce the time constant of the volume conduction path *τ*_v_ and accelerate the decay of charge according to Equations (2)–(3). However, considering the actual product values of resistance and capacitance in Equation (3), according to Table 1, the *τ*_v_ depends mainly on *R*_in_, *C*_in_ and *C*_d_, while the effect of reducing the impedance of the skin–electrode interface (*R*_c_||*C*_c_) is actually small. The *τ*_v_ can be regarded as the discharge time of motion artifacts. While the decrease of the impedance of the skin–electrode interface has little effect on the discharge time of motion artifacts, it has been shown in reference [7] that the decrease of *R*_c_ can reduce the amplitude of motion artifacts. A detailed analysis of the transfer function in Figure 2 shows similar conclusions. 

For the FWEP, the increase of free water content of the material will increase the rate constant of charge escaping into the air *λ* [29], and the increase of *λ* can accelerate the decay of charge according to Equation (5) and Figure 3. 

Generally, when the environmental parameters are constant, the increase of free water content in the textile electrode can accelerate the decay of the charge of the FWEP and make the FWEP play a leading role.

### 3.2. Simulation Experiments

**Figure 4:**
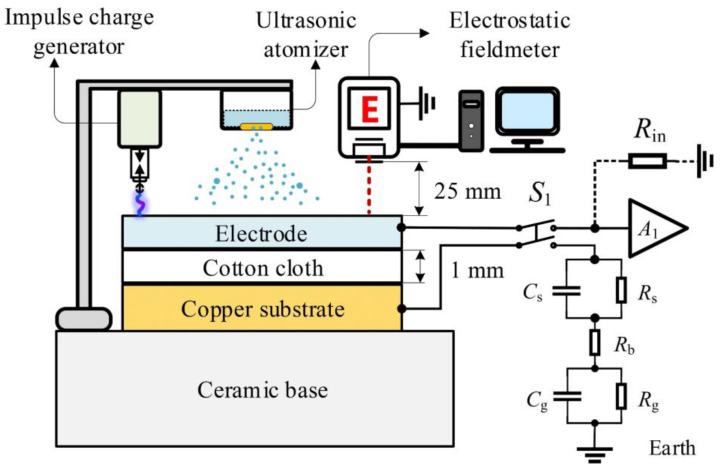
The schematic diagram of the comparative experimental devices. This set of devices can simulate the charge decay of a charged capacitive textile electrode through the volume conduction path and the FWEP separately, and can also simulate the compound action of the both paths.

In order to verify the effect of water on the charge decay of the textile electrode, a series of comparative experiments have been implemented. The schematic diagram of the comparative experimental device is shown in Figure 4, mainly including the impulse charge generator, the ultrasonic atomizer, the electrostatic voltmeter, the ceramic base, the equivalent circuit of the volume conduction path, the copper substrate, the textile electrode and the cotton cloth. The size of the textile electrode is 3 × 4 cm × 0.8 mm, and the material is hydrophilic [32]. The size of the cotton cloth is 4 × 5 cm × 1 mm. When the ultrasonic atomizer works, it can spray the atomized drops onto the textile electrode quantitatively and evenly by controlling the operating time. In the equivalent circuit of the volume conduction path, when the double-pole double-throw (DPDT) switch *S*_1_ closes, the capacitor model of textile electrode, cotton cloth and copper substrate can be equivalent to the equivalent resistance and capacitance of the skin–electrode interface *R*_c_ and *C*_c_. The equivalent circuit of the human side was set up according to Figure 2, and the circuit parameters (*R*_s_, *C*_s_, *R*_g_, *C*_g_ and *R*_b_) were taken from Table 1. The voltage follower *A*_1_ with high input impendence *R*_in_ was set up for simulating a floating ground system in the equivalent circuit of the volume conduction path. 

Under the same environment conditions (25 °C and 65% RH), an impulse charge (d*Q*/d*t* is 27 × 10^−6^ A in *t* being 0.01 s [7]) generated by the impulse charge generator (Haefely PU 12) is injected into the textile electrode in each test. At the same time, the electrostatic voltmeter (SIMCO FMX-004) measures the electrostatic potential on the electrode and records the value every one second.

**Figure 5:**
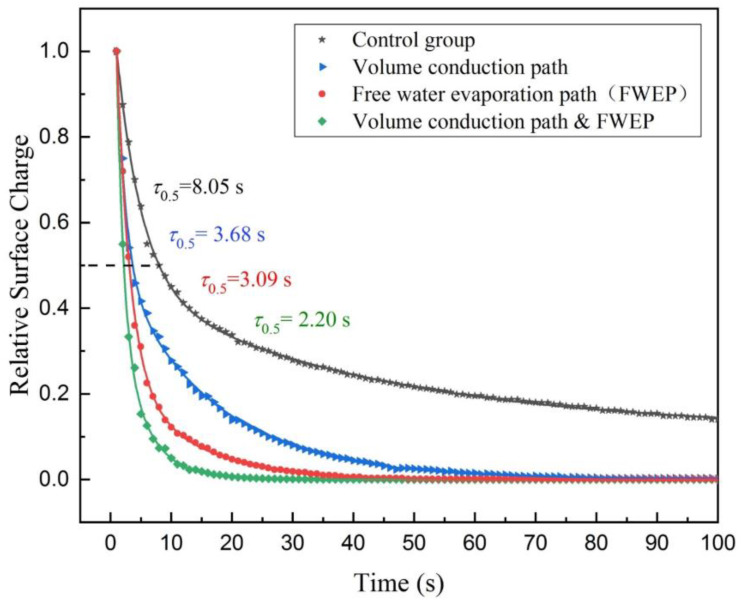
Relative surface charge with time for the textile electrode through different paths by experiments at 25 °C and 65% relative humidity (RH), where the FWEP contributes to the surface charge decay of the textile electrode.

(1) Experiment I (control group): Firstly, dry the moisture of the textile electrode and the cotton cloth in a drying oven. Secondly, turn off the ultrasonic atomizer and open the DPDT switch *S*_1_. Thirdly, inject a pulse charge into the textile electrode by the impulse charge generator and record the electrostatic potential data of the electrostatic voltmeter. Finally, normalize the data and plot these data as the fitting curve with black star data points in Figure 5.

(2) Experiment II (study group, volume conduction path): Firstly, dry the moisture of the textile electrode and the cotton cloth in a drying oven. Secondly, turn off the ultrasonic atomizer and close the DPDT switch *S*_1_. Thirdly, inject a pulse charge into the textile electrode by the impulse charge generator and record the electrostatic potential data of the electrostatic voltmeter. Finally, normalize the data and plot these data as the fitting curve with blue triangle data points in Figure 5.

(3) Experiment III (study group, free water evaporation path): Firstly, dry the moisture of the textile electrode and the cotton cloth in a drying oven. Secondly, turn on the ultrasonic atomizer, keep the operating time of the ultrasonic atomizer for 5 s (spray out about 0.05 g of water) and open the DPDT switch *S*_1_. Thirdly, inject a pulse charge into the textile electrode by the impulse charge generator and record the electrostatic potential data of the electrostatic voltmeter. Finally, normalize the data and plot these data as the fitting curve with red circular data points in Figure 5.

(4) Experiment IV (study group, volume conduction path and free water evaporation path): Firstly, dry the moisture of the textile electrode and the cotton cloth in a drying oven. Secondly, turn on the ultrasonic atomizer, keep the operating time of ultrasonic atomizer for 5 s and close the DPDT switch *S*_1_. Thirdly, inject a pulse charge into the textile electrode by the impulse charge generator and record the electrostatic potential data of the electrostatic voltmeter. Finally, normalize the data and plot these data as the fitting curve with green diamond data points in Figure 5.

It can be seen from Figure 5 that the textile electrode charge decay measured by the experiments demonstrates the exponential decay. When the charge of the textile electrode decays naturally without additional path, the half-life is about 8.05 s as shown by the control group. Compared with the control group, the charge decay can be accelerated by additional paths. The half-life is about 3.68 s through the volume conduction path, while the half-life is about 3.09 s through the FWEP. Under the simultaneous action of the volume conduction path and FWEP, the half-life is 2.20 s. It can be seen that the FWEP contributes to the surface charge decay of the textile electrode.

### 3.3. Effect of the Free Water Content of Textile Electrode on the Charge Decay through the FWEP

Reference [29] shows that the increase of the free water content of insulating textile is helpful to the charge decay through the FWEP by experiments, but whether there is a similar conclusion for conductive textile (textile electrode) needs to be further verified by experiments. Therefore, on the basis of Experiment III, the experiments of charge decay of textile electrode with different free water content have been done. 

**Figure 6:**
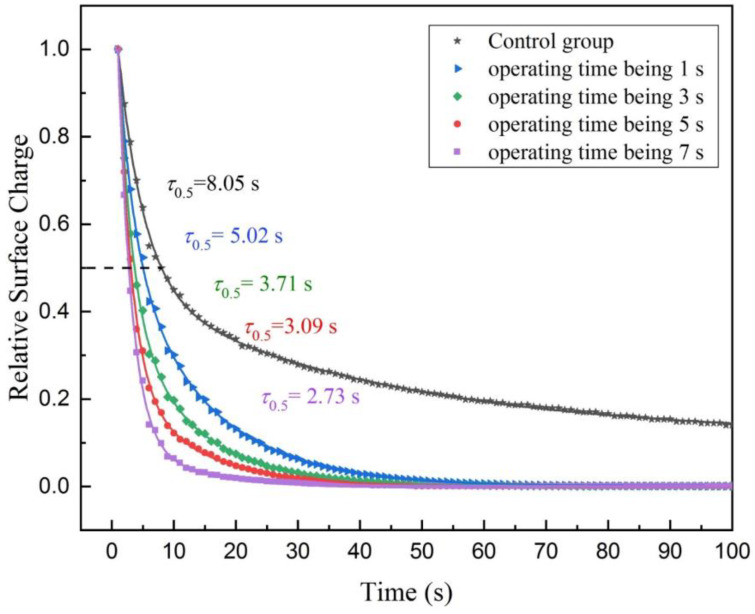
Relative surface charge decay with time for the textile electrode with different free water content through the FWEP at 25 °C and 65% RH. The more free water content of the textile electrode there is, the faster the surface charge decays through the FWEP.

Firstly, it is assumed that the operating time of the ultrasonic atomizer is proportional to the free water content of the textile electrode. On the basis of Experiment III, the free water content of the textile electrode increases in turn by controlling the different operating time of the ultrasonic atomizer (operating time being 1 s, 3 s, 5 s and 7 s, separately), and the electrostatic potential data of the electrostatic voltmeter are recorded, separately, and have been normalized and plotted in Figure 6. The half-life is 5.02 s when the operating time of the ultrasonic atomizer is 1 s. The half-life is 3.71 s when the operating time of the ultrasonic atomizer is 3 s. The half-life is 3.09 s when the operating time of the ultrasonic atomizer is 5 s (using the data of Experiment III). The half-life is 2.73 s when the operating time of the ultrasonic atomizer is 7 s. As shown in Figure 6, the half-life of the surface charge decay of the textile electrode decreases with the increase of the operating time of the ultrasonic atomizer. However, the decrease of the half-life as a function of the operating time of the ultrasonic atomizer is not linear, the decrease trend becomes slow with the further increase of the operating time.

## 4. Controllable Humidification Design of Capacitive Electrode

**Figure 7:**
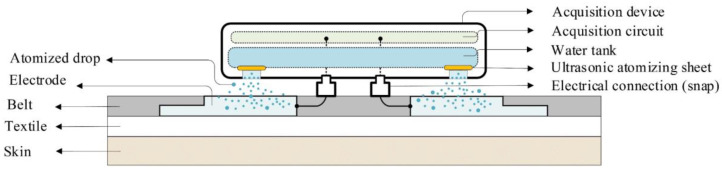
The schematic diagram of the controllable humidification design for a capacitive electrode, showing the working state of the ultrasonic atomization sheets.

According to the theoretical analysis and experiments, the increase of the free water content of the charged textile electrode can accelerate the charge decay through the FWEP. Therefore, a controllable humidification design of a capacitive electrode is proposed for charge decay acceleration and motion artifact reduction. Figure 7 is a schematic diagram of the proposed design, where the electrode with silver fiber is embedded in the belt, and the surface of the electrode is exposed on both sides of the belt for signal acquisition and electrode humidification. As shown in Figure 7, the acquisition device includes an acquisition circuit, a water tank and two ultrasonic atomizing sheets. Through the cavitation effect, the ultrasonic atomizing sheets can oscillate water into plenty of atomized drops. The number of atomized drops can be quantified by adjusting the operating time of ultrasonic atomization sheets (Figure 7 shows the working state of the ultrasonic atomization sheets). The acquisition device and the electrodes on the belt are fixed and electrically connected by the snaps.

**Figure 8:**
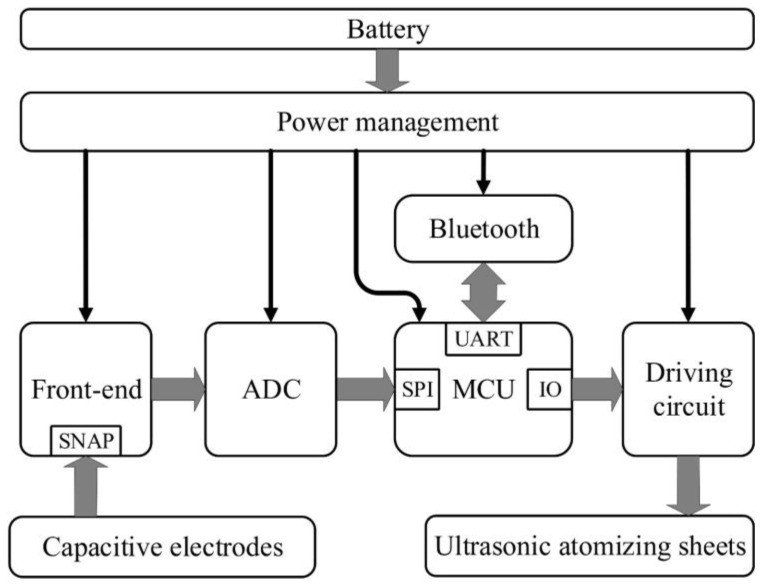
The schematic diagram of the circuit, mainly including the capacitive electrodes, the front end, the analog-to-digital converter (ADC), the micro control unit (MCU), the ultrasonic atomizing sheets, the driving circuit of ultrasonic atomizing sheets, the Bluetooth, the power management and the battery.

The schematic diagram of the acquisition circuit is shown in Figure 8, mainly including the capacitive electrodes, the front end, the analog-to-digital converter (ADC), the micro control unit (MCU), the ultrasonic atomizing sheets, the driving circuit of ultrasonic atomizing sheets, the Bluetooth, the power management and the battery. The capacitive electrodes include two capacitive acquisition electrodes and a C-DRL electrode. The front end is designed according to the proposed structure in reference [40], and a C-DRL circuit for common mode interference suppression is further added. Moreover, the ADC is selected as ADS1292, the MCU is selected as MSP430F5528 to process the data from the ADC, the Bluetooth is selected as CC2540 to transmit data and the power management supplies power to the system through the rechargeable lithium battery. The driving circuit receives the instruction of the MCU and controls the operating time of ultrasonic atomizing sheets to produce quantitative atomized drops. In the actual configuration, the ultrasonic atomizing sheets will work for 5 seconds by default, and can also be controlled by a program according to the requirement.

**Figure 9:**
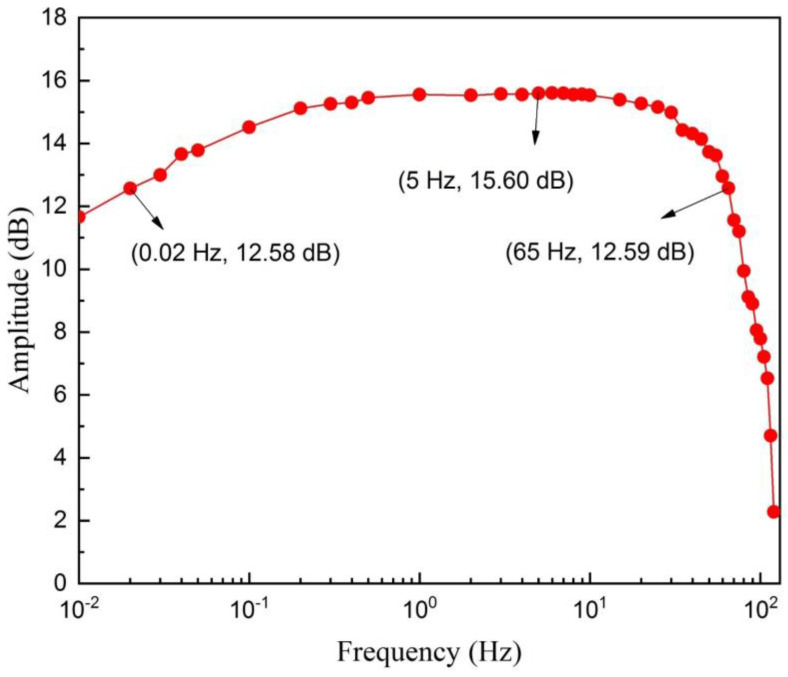
The frequency response of the electrocardiogram (ECG) signal acquisition circuit, where the bandwidth is 0.02 Hz to 65 Hz.

As shown in Figure 9, the bandwidth of the ECG signal acquisition circuit is 0.02 Hz to 65 Hz, which can meet the standard frequency of the ECG signal 0.67–40 Hz [41]. CMRR measurement is carried out under the condition of the common input 50 Hz sinusoidal signal with the amplitude 1000 mV and the differential input 50 Hz sinusoidal signal with the amplitude 2 mV. As shown in Table 2, the CMRR of the system can reach 104.8 dB, which is greater than the international standard IEC 60601-2-47 (78 dB) [42] and the American national standard ANSI/AAMI EC11 (95 dB) [43].

**Figure 10:**
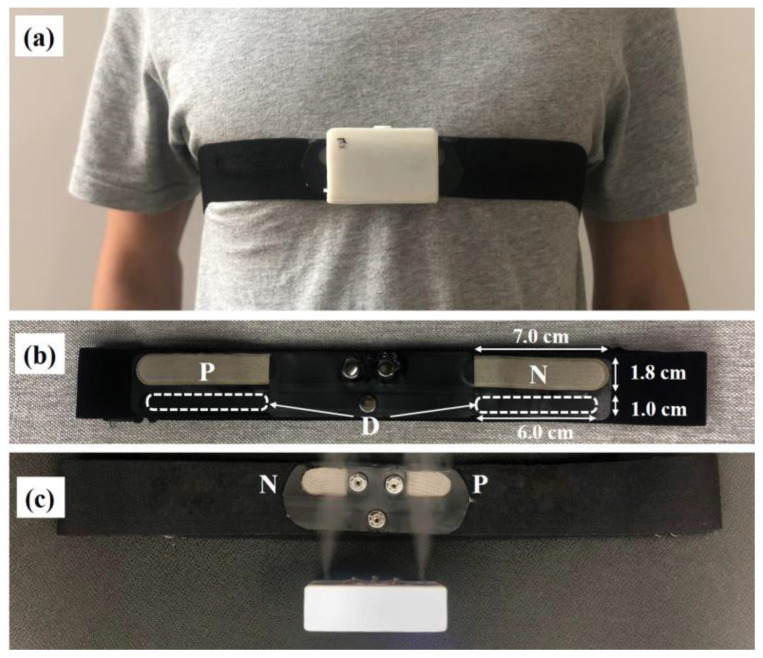
The prototype diagram of the ECG acquisition device, electrodes and belt; (**a**) the capacitive ECG acquisition device is worn; (**b**) the schematic diagram of the acquisition side of the electrodes on the belt; (**c**) the schematic diagram of the humidification side of the electrodes on the belt, where the ultrasonic atomizing sheets are in working state.

Figure 10 is the prototype diagram of the ECG acquisition device, electrodes and belt, where the capacitive ECG acquisition device was worn on the chest in Figure 10a. Figure 10b is the schematic diagram of the acquisition side of the electrodes on the belt, where the capacitive acquisition electrodes P and N (both sizes are 7.0 × 1.8 cm) use hydrophilic conductive textile material [32], while the C-DRL electrode D uses conductive rubber material [44]. The structure of electrode D contains two exposed parts (marked by white dotted boxes) and their sizes are small (both sizes are 6.0 × 1.0 cm) for avoiding the possible impact of C-DRL not considered in Figure 2. It should be mentioned that the role of the capacitive electrode D is to form a negative feedback loop for common mode interference suppression in capacitive ECG acquisition. There are two main reasons for choosing the conductive rubber material. One of the reasons is that the use of hydrophobic conductive rubber material can avoid the atomized drops unexpectedly wetting the electrode D and affecting the experimental results. Another reason is that the conductive rubber material is relatively difficult to deform, which can limit the deformation of the acquisition electrodes to a certain extent, thereby suppressing motion artifacts. Figure 10c is the schematic diagram of the humidification side of the electrodes on the belt, where the acquisition device is removed and the ultrasonic atomizing sheets are in working state.

## 5. Experimental Performance

**Figure 11:**
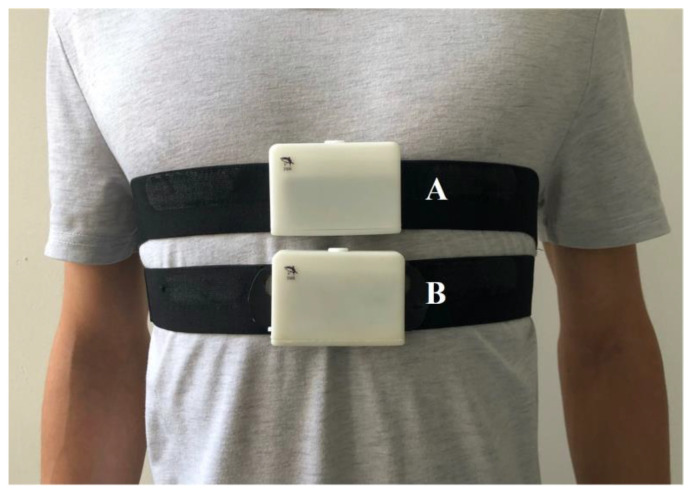
The schematic diagram of the acquisition systems. The humidification part of system **A** can normally work and the humidification part of system **B** has been turned off. These two acquisition systems are used to compare the performance of artifact reduction in the acquisition of the human motion ECG signal between the traditional non-humidified design and the proposed humidified design.

Two identical acquisition systems are used to compare the performance of artifact reduction in the acquisition of a human motion ECG signal between the traditional non-humidified design and the proposed humidified design, as shown in Figure 11. The only difference is that the humidification part of system A will work normally (it will work continuously for 5 s before the beginning of signal acquisition by default) and the humidification part of system B will always be turned off. The human test subject is the volunteer from our group, one male student aged 25 without known cardiac pathology.

**Figure 12:**
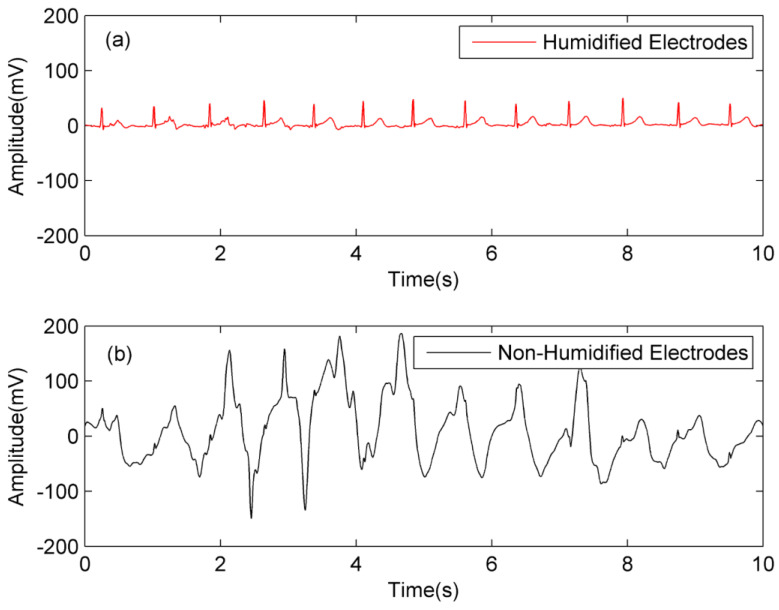
The simultaneous measurement results of human motion ECG acquisition by humidified design and non-humidified design at 25 °C and 25% RH; (**a**) system A with electrodes humidified; (**b**) system B with electrodes non-humidified.

Firstly, the belts with textile electrodes in the above two systems are dried in the drying oven to keep the textile electrodes dry at initial state. Secondly, the belts are placed at the environment with 25 °C and 25% RH for one hour at least. Finally, the volunteer wears these two systems according to Figure 11, and runs at a constant speed of 4 km/h on a treadmill for capacitive ECG signal acquisition in the movement state. The simultaneous measurement results of human motion ECG acquisition by humidified design and non-humidified design were processed by software notch and are plotted in Figure 12.

According to the results of Figure 12, at the low ambient humidity condition (25 °C, 25% RH) and the same state of motion, the ECG signal with the non-humidified electrodes contains motion artifacts too large to recognize P-wave and T-wave clearly, while the one with humidified electrodes has almost no motion artifacts and good waveform characteristics. 

**Figure 13:**
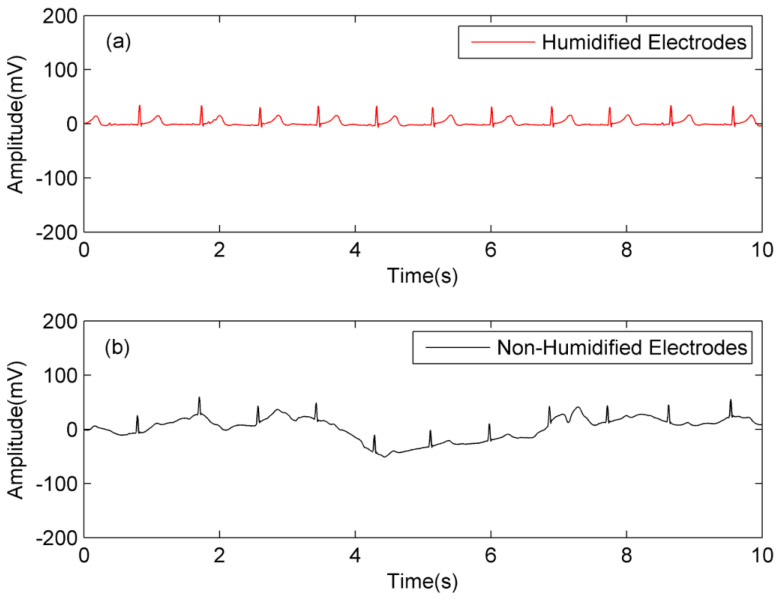
The simultaneous measurement results of human motion ECG acquisition by humidified design and non-humidified design at 25 °C and 65% RH; (**a**) system A with electrodes humidified; (**b**) system B with electrodes non-humidified.

Similar experiments are carried out under the situation with 25 °C and 65% RH and the results are shown in Figure 13. The ECG signal with the non-humidified electrodes still contains some motion artifacts, but the R-wave characteristics are relatively obvious. The ECG signal with humidified electrodes still has almost no motion artifacts and has good waveform characteristics. 

For quantitative comparison, the signal-to-noise ratio (SNR) [45] is defined as:

(6) $$\mathrm{SNR}=10\log\left(\frac{P_{R-\mathrm{peak}}}{P_{\mathrm{noise}}}\right)$$

where P_R-peak_ is the power of 100-ms ECGs centered on the detected R-wave peaks and P_noise_ is the power of the ECGs outside those 100-ms ECGs centered.

Table 3 gives a comparison of SNR between humidified electrodes and non-humidified electrodes at different ambient humidity. As shown in Table 3, with the increase of ambient humidity, the SNRs of all electrodes are improved. At different ambient humidity, the SNR increment of non-humidified electrodes is larger than that of humidified electrodes, which may be regarded as due to the humidified electrodes having obtained sufficient humidity through the humidification process and the effect of ambient humidity being limited. When the ambient humidity is 25% RH, the SNR of humidified electrodes is 33.32 dB higher than that of non-humidified electrodes, and when the ambient humidity is 65% RH, the SNR of humidified electrodes is 22.67 dB higher than that of non-humidified electrodes.

Based on the above quantitative comparison, it can be seen that the proposed design can keep the electrodes moist and avoid the generation of large motion artifacts for obtaining ECG signals with high SNR.

It should be noted that due to the hydrophilicity of the textile electrodes used, the equivalent resistance of the skin electrode interface *R*_C_ in the proposed design may have been reduced after humidification, which will attenuate the amplitude of motion artifacts. Therefore, the results in Figure 12a and Figure 13a may be the simultaneous contribution of the volume conduction path and the FWEP. In any case, the experimental results show that the proposed design can effectively suppress motion artifacts and maintain the stability of signal quality at both low and high ambient humidity.

## 6. Discussion and Conclusions

For wearable capacitive ECG acquisition, capacitive electrodes may cause severe motion artifacts due to the relatively large friction between the electrodes and the dielectrics. In some studies, water can effectively suppress motion artifacts, but these studies lack a complete analysis of how water can suppress motion artifacts, and the role of FWEP is not considered. In this paper, the effect of water on charge decay of textile electrode is studied systematically, and a capacitive electrode controllable humidification design is proposed to suppress motion artifacts. Compared with the existing electrode humidification designs, the proposed electrode humidification design can be controlled by a program to suppress motion artifacts at different ambient humidity, and can be highly integrated for wearable application.

Firstly, the charge decay model of a textile electrode is analyzed. It is pointed out that the FWEP can effectively accelerate the charge decay and suppress the motion artifacts. Secondly, through theoretical analysis and experiment, it is verified that water can accelerate the decay of charge through the FWEP. Thirdly, a capacitive electrode humidification design is proposed. Finally, the performance of the proposed design is verified by the simultaneous measurement results of humidified design and non-humidified design at both low and high ambient humidity, which shows the feasibility of artifact reduction at both low and high ambient humidity. The SNR of humidified design is 33.32 dB higher than that of non-humidified design at 25% RH and is 22.67 dB higher than that of non-humidified electrodes at 65% RH. In general, the proposed design is an effective design to suppress the motion artifacts in capacitive ECG measurements.

In order to achieve a better effect for motion artifact suppression in the proposed design, it is helpful to take the hydrophilic conductor materials with a high rate constant of charge escaping into the air (*λ*) as electrodes, such as cotton blended conductive textile. In the experiments of this paper, the humidification part of the proposed design only works for 5 s before the beginning of acquisition, and does not adjust the humidification time adaptively by monitoring the humidity of the electrode and the numerical value of the motion artifacts. In the future, an effective adapting method for humidification will be studied. In addition, the results of ECG acquisition included the simultaneous contributions of the volume conduction path and the FWEP. For quantitative analysis of contribution by FWEP, it may be effective to cover some conductive and impermeable materials on the acquisition side of the electrodes in the future study.

## Figures and Tables

**Table 1:** The parameter description and values at 20 °C and 65% relative humidity (RH).

Parameter	Value	Meaning
*R*_c_ || *C*_c_	305 MΩ || 34 pF	The equivalent resistance and capacitance of skin–electrode interface (dielectric is cotton) [33]
*R*_s_ || *C*_s_	1 MΩ || 10 nF	The resistance and capacitance of stratum corneum [5,33,34]
*R*_g_ || *C*_g_	10 MΩ || 200 pF	The resistance and capacitance of body against to earth [7,35]
*R*_in_ || *C*_in_	100 GΩ || 5 pF	The equivalent input impedance and capacitance of the acquisition front end based on LMP7702 [33,36]
*R*_b_	1 kΩ	The human body equivalent resistance [33,37]
*C*_d_	200 pF	The distribution capacitance against earth of the floating ground system [35]
*Z*	1.8 × 10^−4^ m^2^/(V·s)	The ion mobility in the air [26]
*n*	2500 #/m^3^	Equilibrium ion concentration at atmospheric pressure at sea level [38]
*ε*_0_	8.85 × 10^−12^ F/m	Vacuum dielectric constant
*λ*	0.2	The rate constant of charge escaping into the air (a similar material value is taken here) [29]

**Table 2:** CMRR measurement result.

Mode	Input Signal		Output Amplitude (mV)	Gain	CMRR (dB)
	Frequency (Hz)	Amplitude (mV)			
Differential	50	2	12.2	6.1	104.8
Common		1000	0.035	3.5 × 10^−5^	

**Table 3:** Comparison of signal-to-noise ratio (SNR) between humidified electrodes with non-humidified electrodes at different ambient humidity.

Ambient Humidity	Status of Electrodes	SNR (dB)
25% RH	Humidified	22.26
	Non-humidified	−11.06
65% RH	Humidified	26.53
	Non-humidified	3.86
